# Structural Equation Modeling of Person-Centered Nursing in Hospital Nurses

**DOI:** 10.3390/healthcare10030514

**Published:** 2022-03-11

**Authors:** Yeon Hee Bae, Hye-Ah Yeom

**Affiliations:** 1 Department of Nursing, Suwon Women’s University of Korea, Suwon 16632, Korea; violin802@swc.ac.kr; 2 Department of Nursing, The Catholic University of Korea, Seocho-gu, Seoul 06591, Korea

**Keywords:** nurses, patient-centered care, empathy, clinical competence, structural equation modeling

## Abstract

Background: This study aimed to develop and test a model of person-centered nursing (PCN) for hospital nurses using Structural Equation Modeling (SEM). Methods: This was a cross-sectional descriptive study. The subjects of this study were 340 clinical nurses in South Korea. A survey was conducted using an online questionnaire. Data were analyzed using SPSS 18.0 and AMOS 21.0. Results: Results of the validity test of the hypothesized model showed that goodness-of-fit indices satisfied the criteria (χ^2^ = 573.767 (df = 257, *p* < 0.001), TLI = 0.92, CFI = 0.93, SRMR = 0.02, RMSEA = 0.06), and 10 of the 15 paths established in the hypothesized model were statistically supported. The model’s explanatory power was 82%, which showed that empathy directly and indirectly affected PCN, and that nursing competency directly affected clinical nurse performance of PCN. In addition, nursing competency, interpersonal relationship ability, and moral sensitivity indirectly influenced PCN through empathy. Conclusions: In order to promote PCN for clinical nurses, it is desirable to develop in-hospital education programs that can improve nursing competency, interpersonal relationship ability, and moral sensitivities, emphasizing elements of empathy. Empathy was an important mediating factor that influenced the relationships between PCN and related variables.

## 1. Introduction

Patient-centered healthcare services are a major concept in the healthcare field [1]. In South Korea, the domain of person-centeredness is included in Korean hospital evaluation criteria [2], and patient experiences with patient-centered care have been assessed by the Health Insurance Review and Assessment Service (HIRA) [3]. Hospitals reflect the concept of person-centered care in their policies and visionary plan [4], and make efforts to maintain their healthcare service environments in ways that are more supportive of patient rights [5]. In clinical nursing, despite the importance of person-centered healthcare services, nurses working at hospitals can experience barriers to person-centered care due to work overload, problems in inter-professional relationships, and/or lack of advanced knowledge in patient care [6,7]. Handling various monitoring devices and advanced medical equipment can limit the time nurses have to build rapport with and perform holistic care for patients [8,9]. Therefore, factors related to person-centered care need to be identified to help nurses implement PCN in their daily practice.

Person-centered nursing (PCN) refers to nursing care that respects a patient as an individual human being and provides holistic care via effective communications [10,11]. PCN is the core of humanistic nursing, as it emphasizes moral values, autonomy, and respect for human dignity [12,13]. By applying PCN, nurses can provide holistic care and increase patient satisfaction with nursing services, which ultimately results in job motivation for nurses [14]. PCN facilitates communications between the patient and the nurse and improves the health status of the patient [15,16,17]. Effective implementation of PCN by nurses is associated with improved quality of nursing services [15], satisfaction with nursing services by the patient’s family [16], and cost- effectiveness by reducing the duration of hospitalization [17]. Therefore, it is important to accommodate strategies for improving nurse competency in PCN.

Previous research has reported the qualitative aspects of PCN [18,19,20] and individual predictors of PCN [16,21,22,23]. Specifically in South Korea, most research on PCN has been conducted in long-term care settings [16,22,23], with sparse focus on hospital nurses. Considering that PCN is influenced by various environmental and individual factors, a hypothesized model of PCN that integrates multiple factors and examines causal pathways among these factors might help increase PCN in a more comprehensive way. Therefore, this study proposes a hypothesized model of PCN in hospital nurses and explores the causal pathways among individual predictors to understand the phenomenon of PCN. 

### 1.1. Research Purpose

This study aimed to develop and test a structural equation model (SEM) of PCN in hospital nurses, and to identify direct or indirect effects of related variables on PCN.

### 1.2. Theoretical Framework for Hypothesized Model 

**Figure 1:**
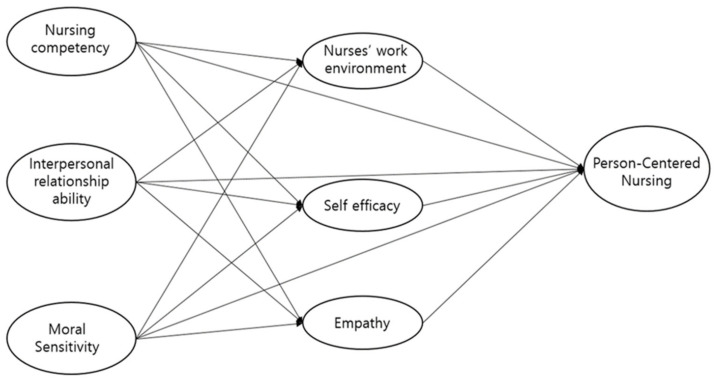
Hypothetical model.

The PCN theory developed by McCormack and McCance [12] was the theoretical framework for the hypothesized model of this study. As a framework that is applicable to complex healthcare systems, PCN theory provides a guide to the person-centered practice of healthcare professionals and focuses on a multi-dimensional process of human-oriented care [12]. The PCN framework is composed of prerequisites (the care environment and the care processes) and the care outcomes. To deliver high-quality care, the prerequisites which are the outer circle of the framework should be primarily established to lead to care outcomes, which are the inner core of the framework and the ultimate goal of the care process. In this study, seven attributes are derived from the PCN framework as study variables, and their causal relationships are hypothesized in the model. Only variables that were reported in previous research as significantly correlated with PCN practice were selected as independent variables in this study. The hypothesized SEM model is presented in Figure 1. 

The variable of nursing competency is one of the prerequisites and was derived from the construct of professional competency in the PCN framework. Nursing competency is known to directly affect PCN and contributes to a desirable nursing work environment [24]. Increased nursing competency is also associated with increased self-efficacy [25] and empathy of nurses [26]. Therefore, in the hypothesized model, nursing competency is set as an exogenous variable that affects the nursing work environment, self-efficacy, empathy, and PCN. 

The variable of interpersonal relationship ability was derived from the construct of developed interpersonal skills in the domain of prerequisites. Interpersonal relationship ability directly affects PCN, and increased interpersonal relationship ability is associated with increased PCN [27]. Nurses’ interpersonal skills contribute to positive nursing work environments and enhance their self-efficacy [28] and empathy [29]. Therefore, interpersonal relationship ability is proposed as an exogenous variable that directly affects nursing work environment, empathy, and PCN in the hypothesized model. 

Another variable derived from the domain of prerequisites is moral sensitivity, which can correspond to the construct of clarity of beliefs and values in the PCN framework. Moral sensitivity refers to a nurse’s awareness of moral value and the moral impact of a nurse’ behavior on a patient [30,31]. Moral sensitivity directly affects PCN and helps build an ethical nursing work climate [21]. Increased moral sensitivity is associated with increased self-efficacy [32] and empathy [33]. Therefore, in the hypothesized model, moral sensitivity is proposed as an exogenous variable that affects the nursing work environment, self-efficacy, empathy, and PCN. 

The variable of nursing work environment was derived from the construct of physical environment in the domain of care environment in the PCN theory. The nursing work environment refers to physical environments that facilitate or limit professional nursing practice [34]. A poor nursing work environment is a known barrier to PCN [21] and affects the quality of PCN [22,24]. In this study, the nursing work environment is set as a mediating variable that directly affects PCN in the causal pathway of the hypothesized model. 

The variable of self-efficacy was derived from the construct of potential for innovation and risk-taking in the domain of care environment in the PCN framework. Self-efficacy refers to an individual’s personal belief in completing tasks successfully [35]. Previous research has shown that those with higher self-efficacy are likely to have higher potential for innovation and risk-taking [36,37]. Therefore, in this study, self-efficacy was assumed to be related to the potential for innovation and risk-taking. Increased self-efficacy is associated with a greater level of PCN in nurses [38]. As a mediating variable in the hypothesized model, self-efficacy is assumed to have a direct effect on PCN.

The variable of empathy was derived from the construct of sympathetic presence in the domain of care processes in the PCN framework. Empathy refers to one’s ability to understand the patient’s emotions and take action to support the patient [39]. Increased empathy is associated with higher levels of PCN [40]. In the hypothesized model, empathy is proposed as a mediating variable that directly affects PCN in the causal pathway. Previous research has shown that the nursing work environment is not correlated with self-efficacy [41] and empathy [42]; thus, in the hypothesized model, these three variables are assumed to not affect each other.

## 2. Methods

### 2.1. Study Design

This was a cross-sectional descriptive study designed to develop and test a hypothesized model for PCN in hospital nurses using SEM.

### 2.2. Study Participants

The study participants were 350 nurses who agreed to participate in the study. The inclusion criteria for study participants were adults who were aged 19 or older, understood the purpose of the study and agreed to participate, and were working in hospitals with more than 100 beds. The exclusion criteria for study participants were nurses who did not agree to participate in this study and were not employed in hospitals at the time of data collection. The sample sized met the recommended criteria for SEM research; 200 or more is generally accepted as the minimum sample size for maximum likelihood estimation [43,44]. After excluding 10 incomplete surveys, the final data included 340 subjects, with a 2.9% data loss rate.

### 2.3. Measurements

#### 2.3.1. Person-Centered Nursing

PCN was measured using the Person-Centered Care Assessment Tool (P-CAT) developed by Edvardsson et al. [45] and translated into Korean by Tak et al. [46]. The P-CAT is a 13-item scale, and exploratory factor analysis (EFA) resulted in the two factors of individualized care and individual patient information sharing being derived. The item example of the PCN instrument is “I include the value and perspective of the subject when planning care”. Each item was measured on a five-point Likert scale, with a higher score indicating a greater degree of PCN. In this study, Cronbach’s alpha reliability of the scale was 0.72. For the subdomains, Cronbach’s alpha reliability was 0.67 for the domain of individualized care and 0.58 for the domain of individual patient information sharing.

#### 2.3.2. Nursing Competency

Nursing competency was measured using the nursing competency scale developed by Jang [47] and revised by Kim [48]. This is a 13-item scale composed of four subdomains: empirical competency, ethical competency, personal competency, and esthetic competency. Specifically, esthetic competency refers to a nurse’s ability to provide patient-oriented care through understanding and supportive clinical judgment for the patient. The EFA showed that the factor loadings for all items were greater than 0.4, so none of them were deleted. The item example of the nursing competency instrument is “I prepare individual nursing alternatives to the complex nursing problems and needs of patients and families, and give patients hope and new possibilities for life”. Each item ranged from 1 (very unlikely) to 4 (very likely), and a higher score indicated a higher level of nursing competency. In this study, Cronbach’s alpha reliability of the scale was 0.88. For the subdomains, Cronbach’s alpha reliability was 0.75 for empirical competency, 0.64 for ethical competency, 0.66 for personal competency, and 0.78 for esthetic competency.

#### 2.3.3. Interpersonal Relationship Ability

Interpersonal relationship ability was measured using the Korean version Relationship Change Scale (RCS) [49,50,51]. The RCS is an 18-item scale composed of six subdomains: communication, trust, intimacy, sensitivity, openness, and understanding. The EFA showed that the factor loadings for all items were greater than 0.4, so none of them were deleted. The item example of the interpersonal relationship ability instrument is “I tend to keep my relationship with others satisfactory”. Each item was measured on a five-point Likert scale, with a higher score indicating a greater degree of interpersonal relationship ability. In this study, Cronbach’s alpha reliability of the scale was 0.89. For the subdomains, Cronbach’s alpha reliability was 0.68 for communication, 0.62 for trust, 0.78 for intimacy, 0.83 for sensitivity, 0.67 for openness, and 0.77 for understanding.

#### 2.3.4. Moral Sensitivity

Moral sensitivity was measured using the Korean Moral Sensitivity Questionnaire (K-MSQ) [52,53]. The K-MSQ is a 27-item scale composed of five subdomains: patient-oriented care, professional responsibility, conflict, meaning, and benevolence. Of these, the subdomains of conflict, meaning, and benevolence were deleted because the factor loadings for items in these domains were <0.4. This left two subdomains, patient-oriented care (5 items) and professional responsibility (7 items), in the scale. The item example of the moral sensitivity instrument is “I often contemplate my own values and norms that can influence my behavior”. Each item was measured on a seven-point scale, with a higher score indicating a greater degree of moral sensitivity. In this study, Cronbach’s alpha reliability of the scale was 0.88. For the subdomains, Cronbach’s alpha reliability was 0.79 for patient-oriented care and 0.81 for professional responsibility.

#### 2.3.5. Nurses’ Work Environment

Nurses’ work environment was measured using the Korean version Practice Environment Scale of the Nursing Work Index (PES-NWI) developed by Lake [54] and translated into Korean by Cho et al. [55]. The PES-NWI is a 29-item scale composed of five subdomains: staffing and resource adequacy, nurse–physician relations, nurse manager leadership and support, nursing foundations, and nurse participation. The EFA showed that the factor loadings for all items were greater than 0.4, so there were no deletions. The item example of the nurses’ work environment instrument is “There is a lot of time to spend with patients because there is sufficient support service”. Each item was measured on a four-point Likert type scale, with a higher score indicating a more positive perception of work environment. In this study, Cronbach’s alpha reliability of the scale was 0.93. For the subdomains, Cronbach’s alpha reliability was 0.80 for staffing and resource adequacy, 0.83 for nurse–physician relations, 0.77 for nurse manager leadership and support, 0.80 for nursing foundations, and 0.85 for nurse participation.

#### 2.3.6. Self-Efficacy

Self-efficacy was measured using the General Self-Efficacy Scale [56]. The scale consists of 17 items, and the EFA showed that the factor loadings for all items were greater than 0.4, so there were no deletions. Three factors were conceptualized as willingness, effort, and confidence. The item example of the self-efficacy instrument is “I’m sure I can do it when I plan something”. Each item was measured on a five-point Likert scale, with a higher score indicating a greater degree of self-efficacy. In this study, Cronbach’s alpha reliability of the scale was 0.95. For the subdomains, Cronbach’s alpha reliability was 0.91 for willingness, 0.88 for effort, and 0.81 for confidence.

#### 2.3.7. Empathy

Empathy was measured using the Compassionate Competence Scale (CCS) [57]. The CCS is a 17-item scale composed of three subdomains: communication, sensitivity, and insight. The EFA showed that the factor loadings for all items were greater than 0.4, so none of them were deleted. The item example of the empathy instrument is “I can express empathy for the subject through communication”. Each item was measured on a five-point Likert scale, with a higher score indicating a greater degree of empathy. In this study, Cronbach’s alpha reliability of the scale was 0.91. For the subdomains, Cronbach’s alpha reliability was 0.85 for communication, 0.84 for sensitivity, and 0.69 for insight.

### 2.4. Data Collection Procedure

This study was conducted with the approval of the Institutional Review Board of C University in South Korea (IRB NO. MC20QESI0062). The data were collected in July 2020. Participants were recruited online using an internet domain that provides health and job information for Korean nurses (www.nurscape.net/ accessed on 11 July 2020). After obtaining permission from the chief executive officer of the domain regarding subject recruitment for the study, researchers uploaded a recruitment flyer and a URL link for the online survey to the site. After reading about the purpose and data collection process of the study, individuals who were willing to participate completed an online informed consent form and participated in the online survey on a voluntary basis. Personal identification information, such as name or social security number, was not collected. All data were solely used for research purposes. The survey took about 15 min to complete, and the participants were compensated the equivalent of $5 for their participation.

### 2.5. Data Analysis

The data were analyzed using the SPSS 18.0 and AMOS 21.0 programs. Descriptive statistics were produced for all study variables. The validity and reliability of the scales were examined through EFA and Cronbach’s alpha reliabilities. The normality of the hypothesized model was tested for skewness and kurtosis, and correlations among the measured variables were evaluated using Pearson’s correlation coefficient. Model fit of the latent variables was examined by confirmatory factor analysis (CFA), average variance extracted (AVE), and construct reliability (CR). Fit of the data to the measured and hypothesized models was assessed by fit indices, including chi-square (χ^2^), normal chi-square (χ^2^/df), Tucker–Lewis index, comparative fit index (CFI), standardized root mean square residual (SRMR), and root mean square error of approximation (RMSEA). The bootstrapping method was used to examine the significance of the direct effects and the specific indirect effects of the hypothesized model in the multiple medication analysis.

## 3. Results

### 3.1. General Characteristics of Participants

The majority of the study participants were women (93.5%) younger than 30 years (85.6%). More than half of the respondents were single (60.9%) baccalaureate degree graduates (69.7%), and were working at general hospitals with more than 500-bed units (67.6%). In their job positions, 81.5% were staff nurses and 41.2% were working in general wards. The majority of the participants (71.8%) had clinical experience of 10 years or less (Table 1).

### 3.2. Descriptive Statistics and Normality of Measured Variables

Descriptive statistics of the measured variables are presented in Table 2. Normality analysis showed that absolute values of skewness and kurtosis of all variables did not exceed 1.97 and 2.58, respectively, meeting the recommended criteria for a normal distribution [58] (Table 2).

### 3.3. Goodness-of-Fit of Measurement Model

#### 3.3.1. Confirmatory Factor Analysis of Measurement Variables

CFA of the measurement variables showed that the standardized factor loadings (range: 0.62–0.92) and AVE (range: 0.66–0.89) of all variables were 0.5 or higher, supporting the convergent validity of the variables. The highest correlation coefficient between the measurement variables was 0.65, showing conceptual independence among the variables. Discriminant validity was also achieved, as the AVE values were greater than the squared values of the correlation coefficients. The CR values of the measurement variables were ≥0.7 (range: 0.80–0.96), demonstrating internal consistency of the variables.

#### 3.3.2. Model Fit of the Measurement Model

The fitness indices of the measurement model met the recommended criteria (χ^2^ = 633.125 (df = 254, *p* < 0.001), TLI = 0.91, CFI = 0.92, SRMR = 0.02, RMSEA = 0.06).

### 3.4. Model Fit of the Hypothesized Model

In this study, the fitness indices of the hypothesized model met the recommended criteria (χ^2^ = 573.767 (df = 257, *p* < 0.001), TLI = 0.92, CFI = 0.93, SRMR = 0.02, RMSEA = 0.06), so the overall fit of the model was regarded as acceptable. This indicated that the hypothesized model of PCN for hospital nurses was appropriate [43,59,60].

### 3.5. Path Analysis for the Hypothetical Model

**Figure 2:**
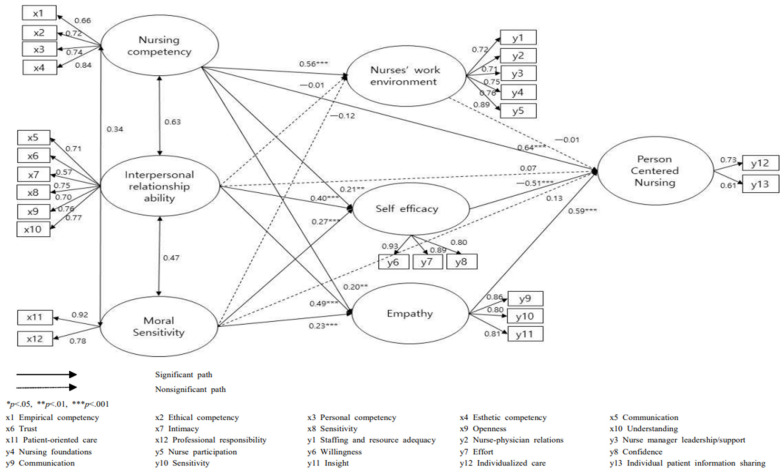
Path diagram of the hypothetical model.

Analysis of the pathways showed that 10 of 15 pathways were statistically significant (Figure 2). The error variances reported in the path diagram were used to indicate interrelationship between variables in this study. Since the correlations between independent variables is a default in SEM statistics, direct and indirect effects in the pathways were used to represent the strength of the correlation between study variables (Table 3). There were significant direct effects of nursing competency (β = 0.64, *p* < 0.001), self-efficacy (β = −0.51, *p* < 0.001), and empathy (β = 0.59, *p* < 0.001) on PCN. Nursing competency (β = 0.56, *p* < 0.001) had a direct effect on nurses’ work environment. Nursing competency (β = 0.21, *p* = 0.001), interpersonal relationship ability (β = 0.40, *p* < 0.001), and moral sensitivity (β = 0.27, *p* < 0.001) had significant direct effects on self-efficacy. In terms of empathy, nursing competency (β = 0.20, *p* = 0.002), interpersonal relationship ability (β = 0.49, *p* < 0.001), and moral sensitivity (β = 0.23, *p* < 0.001) showed direct effects on empathy.

Analysis of specific indirect effects showed that nursing competency (β = −0.12, *p* = 0.001), interpersonal relationship ability (β = −0.19, *p* = 0.001), and moral sensitivity (β = −0.10, *p* = 0.001) had indirect effects on PCN via self-efficacy. There were also significant indirect effects of nursing competency (β = 0.13, *p* = 0.001), interpersonal relationship ability (β = 0.26, *p* = 0.001), and moral sensitivity (β = 0.10, *p* = 0.001) on PCN via empathy.

## 4. Discussion

This study developed the SEM of PCN for hospital nurses based on the PCN framework [12] and examined the direct and indirect effects of independent variables on PCN. In the SEM, 82% of the variance of the PCN was explained by the related variables, and the model fit indices met the recommended criteria, showing that the hypothesized model was appropriate to explain PCN in hospital nurses.

In this study, an important factor in the PCN of hospital nurses was empathy. Unlike other independent variables, empathy reaches PCN through three variables (nursing competency, interpersonal relationship ability, moral sensitivity), implying its conceptual influence on the PCN of hospital nurses. The crucial role of empathy in PCN has been recognized [40], as it helps nurses understand the patient in a more comprehensive way [61]. An empathy-based relationship between the nurse and the patient can decrease health problems of the patient and accommodate a healthy lifestyle pattern of the patient [62]. Various components of empathy (emotional, cognitive, moral, and relationship elements) have been reported [63]. In designing future empathy interventions, the multi-dimensional aspects of empathy should be incorporated into education programs to enhance more positive outcomes in PCN practice among hospital nurses.

In this study, increased empathy was associated with greater levels of nursing competency, interpersonal relationship ability, and moral sensitivity, which is consistent with the findings of previous research [29,64,65,66]. Empathy is a vital component of inter-personal relationships and care processes [67,68] and can be facilitated through training interventions [69,70]. Because lack of empathy is a barrier to ethical nursing practice [71] and PCN [40], education programs for nurses should contain empathy as a major component, and specific strategies for improving empathy should be built into future PCN intervention programs.

In the SEM, the second most influential factor on PCN was nursing competency, which is consistent with the view that nurse competency leads to quality nursing care [72]. It has been reported that nursing competency is an attribute of effective work performance of nurses in the clinical context [73]. Therefore, to implement a high level of PCN at hospitals, the nursing competency of individual nurses needs to be enhanced along with institutional support for PCN. In this study, although the overall concepts of self-efficacy and nursing competency were assumed to be considerably associated, they were only weakly related. Nursing competency was measured by four subdomains (empirical competency, ethical competency, personal competency, and esthetic competency) and self-efficacy was measured by three subdomains (willingness, effect, and confidence). Their subdomains could be mutually exclusive, which may have resulted in the weak relationship between the two variables. To understand the influence of nursing competency on self-efficacy more specifically, the nature of their relationships needs to be further examined in future replication studies.

Interestingly, the nature of the relationship between self-efficacy and PCN differed by data analysis approach. Whereas self-efficacy was positively associated with PCN in the univariate correlation analysis, self-efficacy affected PCN negatively in the SEM. It is assumed that statistical causality among variables is due to the correlations among multiple variables in the SEM in an integrated way [43], which might have impacted the relationship between self-efficacy and PCN in this study. In addition, the self-efficacy measured in this study was general self-efficacy, which might reflect a personal trait rather than task-specific efficacy in a clinical context. Considering a possible mediating pathway between self-efficacy and PCN, the complex nature of the relationship between work-related self-efficacy and PCN needs to be further examined in future SEM studies. In this study, there were several non-significant pathways in the SEM. PCN was not directly affected by inter-personal skills, moral sensitivity, and nursing work environment. Although these variables were proposed to have direct effects on PCN in the hypothesized model, they were found to have non-significant direct paths with PCN in the path diagram. Considering that SEM analyzes inter-correlations among variables in a comprehensive way, results on pathways could differ by the conceptual nature of the independent variables included in the model. Therefore, the non-significant findings need to be further examined in the future replication studies and SEM models with difference set of PCN predictors as well

In summary, it was found that the PCN of hospital nurses was affected by nursing competency, interpersonal relationship ability, moral sensitivity, and empathy. PCN programs for hospital nurses reported by previous research consisted of self-awareness, interpersonal skills, peer support, and nursing job satisfaction [28] Since nursing competency, moral sensitivity, and empathy are found to affect PCN in this study, previous PCN programs can be expanded by including these components based on the results of this study.

## 5. Limitations

There are several limitations in this study. Because the study participants were recruited from various hospitals, they might have experienced different clinical cultures and systems, indicating the possible involvement of unmeasured external variables. Convenience sampling method was used to recruit study participants for online survey, which may have resulted in a selection bias in hospital size and ability of internet access among nurses. The data were collected online, so there could have been missing values or incomplete responses. In addition, because the study participants were not met in person, it was difficult to assess whether they met the sample selection criteria. Use of a context- or task-specific self-efficacy scale should also be considered to investigate the interplay between self-efficacy and the PCN of hospital nurses in further research. Whereas confidence intervals are an important indicator, the *p*-value was also appropriate data in verifying the direct or indirect effect in the SEM [74]. Consideration of confidence interval along with *p*-value might be meaningful for understanding the SEM data more clearly in future studies.

## 6. Implications

Regardless of these limitations, this study has several implications from the standpoint of nursing theory and practice. From theoretical perspectives, the findings of this study expand the scope of the PCN framework [12] into clinical practice for hospital nurses by comprehensively examining the relationships among PCN-related variables using SEM. The PCN model of hospital nurses can be used as a basis for further research that examines PCN in the community setting. From the standpoint of nursing practice, this study provides strategies for development of PCN education programs for hospital nurses by identifying the essential attributes that influence their PCN. The PCN of hospital nurses is directly affected by four components: empathy, nursing competency, interpersonal relationship ability, and moral sensitivity. These results can be used as evidence for developing PCN education programs for nurses.

## 7. Conclusions

The study results showed that the PCN of hospital nurses was directly affected by empathy, nursing competency, interpersonal relationship ability, and moral sensitivity. Empathy was an important mediating factor that influenced the relationships between person-centered care and related variables of nursing competency, interpersonal relationship ability, and moral sensitivity. Further research should focus on repeated validation of these study findings and examination of the effects of an empathy-based nursing intervention program on improving the PCN of hospital nurses.

## Figures and Tables

**Table 1:** General Characteristics of the Subject (*N* = 340).

Variables	Categories	*N* (%)	Mean ± SD
Gender	Male	22 (6.5)	
	Female	318 (93.5)	
Age (years)	20–29	156 (45.9)	32.29 ± 7.11
	30–39	135 (39.7)	
	40–49	39 (11.5)	
	≥50	10 (2.9)	
Religion	Christian	82 (24.1)	
	Catholic	60 (17.6)	
	Buddhism	17 (5.0)	
	Atheism	181 (53.2)	
Marital status	Single	207 (60.9)	
	Married	129 (37.9)	
	divorce	4 (1.2)	
Educational level	3-year diploma	49 (14.4)	
	4-year BSN	237 (69.7)	
	Master or higher	54 (15.9)	
Number of beds at workplace	100–299	70 (20.6)	
	300–499	40 (11.8)	
	≥500	230 (67.6)	
Hospital location	Seoul province	171 (50.3)	
	Gyeonggi province	66 (19.4)	
	Incheon province	60 (17.6)	
	Gyeongsang province	19 (5.6)	
	Jeolla province	17 (5.0)	
	Chungcheong province	7 (2.1)	
Job Position	Staff Nurse	277 (81.5)	
	Charge nurse	28 (8.2)	
	Nurse administrator/Head nurse	8 (2.4)	
	Nurse Specialist	27 (7.9)	
Working unit	Medical Unit	87 (25.6)	
	Surgical Unit	53 (15.6)	
	Gynecological and Pediatric Unit	26 (7.6)	
	Intensive Care Unit	49 (14.4)	
	Emergency Unit	35 (10.3)	
	Operating and Anesthesia Unit	23 (6.8)	
	other	67 (19.7)	
Work shift	Shift work	233 (68.5)	
	Fixed day shift	107 (31.5)	
Years of nursing experience	<5	127 (37.4)	
	5–10	117 (34.4)	
	≥10	96 (28.2)	
Work experience of current department (years)	<5	227 (66.8)	
	5–10	79 (23.2)	
	≥10	34 (10.0)	

**Table 2:** Descriptive Statistics of Measured Variable (*N* = 340).

Latent Variables	Measured Variables	Range	Mean ± SD	Skewness	Kurtosis
Nursing competency	Empirical competency	1~4	2.56 ± 0.53	−0.13	0.31
	Ethical competency		2.81 ± 0.50	−0.72	1.50
	Personal competency		2.80 ± 0.50	−0.50	1.98
	Esthetic competency		2.69 ± 0.46	−0.34	1.14
Interpersonal relationship ability	Communication	1~5	3.59 ± 0.60	−0.95	2.33
	Trust		3.57 ± 0.61	−0.69	1.70
	Intimacy		3.72 ± 0.62	−0.83	1.53
	Sensitivity		3.70 ± 0.68	−0.57	0.78
	Openness		3.42 ± 0.59	−0.24	0.75
	Understanding		3.68 ± 0.65	−0.67	1.28
Moral Sensitivity	Patient-oriented care	1~7	5.60 ± 0.79	−0.65	0.43
	Professional responsibility		5.64 ± 0.76	−0.65	0.55
Nurses’ work environment	Staffing and resource adequacy	1~4	2.11 ± 0.63	0.16	−0.50
	Nurse–physician relations		2.59 ± 0.64	−0.40	0.18
	Nurse manager leadership/support		2.53 ± 0.61	−0.27	−0.26
	Nursing foundations		2.67 ± 0.50	−0.21	0.41
	Nurse participation		2.24 ± 0.53	0.02	−0.05
Self-efficacy	Willingness	1~5	3.71 ± 0.61	−0.43	1.14
	Effort		3.65 ± 0.62	−0.41	0.95
	Confidence		3.74 ± 0.61	−0.71	1.43
Empathy	Communication	1~5	3.65 ± 0.55	−0.38	1.22
	Sensitivity		3.86 ± 0.57	−0.89	2.33
	Insight		3.54 ± 0.56	−0.24	1.42
Person-Centered Nursing	Individualized care	1~5	3.44 ± 0.59	−0.61	1.54
	Individual patient information sharing		2.81 ± 0.69	0.10	0.01

**Table 3:** Standardized direct, indirect effects for the Hypothetical model.

Independent Variable (a)	Parameter (b)	Dependent Variable (c)	Direct Effect (*p*) (a -> b)	Direct Effect (*p*) (a -> c)	Direct Effect (*p*) (b -> c)	Specific Indirect Effect (*p*) (a- > b- > c)
Nursing competency	Nurses’ work environment	Person-Centered Nursing	0.56 (<0.001)		−0.01 (0.843)	−0.00 (0.961)
Interpersonal relationship ability			−0.01 (0.898)			0.00 (0.953)
Moral Sensitivity			−0.12 (0.065)			0.00 (0.744)
Nursing competency	Self-efficacy	Person-Centered Nursing	0.21 (0.001)		−0.51 (<0.001)	−0.12 (0.001)
Interpersonal relationship ability			0.40 (<0.001)			−0.19 (0.001)
Moral Sensitivity			0.27 (<0.001)			−0.10 (0.001)
Nursing competency	Empathy	Person-Centered Nursing	0.20 (0.002)		0.59 (<0.001)	0.13 (0.001)
Interpersonal relationship ability			0.49 (<0.001)			0.26 (0.001)
Moral Sensitivity			0.23 (<0.001)			0.10 (0.001)
Nursing competency		Person-Centered Nursing		0.64 (<0.001)		
Interpersonal relationship ability				0.07 (0.487)		
Moral Sensitivity				0.13 (0.067)		

## Data Availability

The data presented in the study are only available on request from the corresponding author. The data are not publicly available due to that the study data are part of Doctoral dissertation of the Dr. Yeon Hee Bae, the first author of the study.
